# Development and Psychometric Testing The Suicide Risk Management Efficacy Scale

**DOI:** 10.1192/j.eurpsy.2023.2374

**Published:** 2023-07-19

**Authors:** S. Öztürk, D. Hiçdurmaz

**Affiliations:** 1 Psychiatric Nursing, Hacettepe University Nursing Faculty, Ankara, Türkiye

## Abstract

**Introduction:**

Current suicidology literature emphasizes the need for suicide prevention and awareness training that include specific approaches tailored for specific professional groups. However, it is necessary that the scales used in the assessment of trainings contain the needs of the evaluated groups on risk management. Cancer patients are one of the groups with a high risk of suicide. Studies show oncology nurses have difficulties in recognising and managing the risk of suicide in oncology patients(Granek et al. Psycho-Oncology 2018; 27(1) 148–154, Öztürk and Hiçdurmaz. Journal of Clinical Nursing 2022; 1-15). Also, these studies underline the need to create training programmes peculiar to oncology that increase oncology nurses’ awareness, knowledge, skills and efficacy in recognising and managing suicide risk. However, no study in the current literature presents scale that can assess the effect of these trainings on efficacy of oncology nurses or other professional who work oncology in the management of suicide risk. Valid and reliable scale is required to assess oncology nurses’ efficacy in suicide risk management.

**Objectives:**

This study aim to to develop and test the psychometric properties of the Suicide Risk Management Efficacy Scale (SRMES)

**Methods:**

The study was conducted in two stages: (1) the creation of conceptual frameworks and scale items (2) assessing the scale psychometric properties. At the end of the feedback from 10 experts (Psychiatrists and Psychiatric Nurses), the scale content validity was completed and the scale was applied oncology nurses sample. Data were collected using a Descriptive Characteristic Form and the 26-item SRMES. Data were obtained from 234 oncology nurses. Exploratory, confirmatory factor analyses and reliability analyses were performed.

**Results:**

Exploratory factor analyses extracted a unifactorial solution. Confirmatory factor analysis revealed that the unifactorial model presented highly satisfied and acceptable fit indexes (CMIN/df=1,927; CFI=.94; GFI=.844; IFI=0,941; TLI=.928; RMSEA=.063: NFI=0,884; RFI=0,86). According to the results of the principal component analysis, factor loads in the unifactorial structure are between 0.534 and 0.843. Cronbach’s alpha value of the scale was 0,96, the inter-class reliability coefficient is 0.928.

**Conclusions:**

The result of exploratory factor analysis and confirmatory factor analysis results were satisfactory. The SRMES is a valid and reliable scale that can be used to assess oncology nurses’ efficacy perception on suicide risk management. The SRMES can also be used to evaluate the efficacy perceptions of other health professionals on suicide risk management who perform psycho-social assessments similar to nurses in oncology.

**Disclosure of Interest:**

None Declared

